# Domestic Summary

**Published:** 1838-03-28

**Authors:** 


### DOMESTIC SUMMARY.

NEW WORKS.—The Treatise on the Management and Diseases of Children, by Evanson and Maunsell, lately published in the Select Medical Library, is a valuable work.—Muehry's interesting and entertaining Observations on the Comparative State of Medicine in France, England, and Germany, have been recently translated from the German, by Edward G. Davis, M. D. of Philadelphia, to whom we are indebted for other valuable contributions from the medical literature of that language.—The Final Report of the Committee of the Medical Society, on Dr. Chase's instruments for the radical cure of Hernia, has been republished in a neat volume. The superiority of Dr. Chase's instruments appears to be a surgical point generally conceded.—We have received two very interesting lectures on Lithotomy, delivered at the New York Hospital, by Dr. A. H. Stevens, which we propose noticing at length in our next.

---

*Jefferson Medical College*.—The number of Graduates in this institution for the present year is one hundred and three.

---

Dr. Coates states to us that, as in his operation for erectile tumour, the *threads* were omitted, in compliance with the recommendation of Professor Lallemand, the process he employed does not seem to him to be identical with that of his friend, Dr. Pancoast. When the case is more advanced he means to publish the particulars.

